# FACTORS ASSOCIATED WITH THE LIKELIHOOD OF FURTHER MOVEMENT AMONG MOBILE FEMALE SEX WORKERS IN INDIA: A MULTINOMIAL LOGIT APPROACH

**DOI:** 10.1017/S0021932015000267

**Published:** 2015-08-10

**Authors:** Dipak Suryawanshi, Varun Sharma, Niranjan Saggurti, Shalini Bharat

**Affiliations:** * School of Health Systems Studies, Tata Institute of Social Sciences, Mumbai, India; † HIV and AIDS Program, Population Council, New Delhi, India

## Abstract

Female sex workers (FSWs) are vulnerable to HIV infection. Their socioeconomic and behavioural vulnerabilities are crucial push factors for movement for sex work. This paper assesses the factors associated with the likelihood of movement of sex workers from their current place of work. Data were derived from a cross-sectional survey conducted among 5498 mobile FSWs in 22 districts of high in-migration across four states in southern India. A multinomial logit model was constructed to predict the likelihood of FSWs moving from their current place of work. Ten per cent of the sampled mobile FSWs were planning to move from their current place of sex work. Educational attainment, marital status, income at current place of work, debt, sexual coercion, experience of violence and having tested for HIV and collected the results were found to be significant predictors of the likelihood of movement from the current place of work. Consistent condom use with different clients was significantly low among those planning to move. Likewise, the likelihood of movement was significantly higher among those who had any STI symptom in the last six months and those who had a high self-perceived risk of HIV. The findings highlight the need to address factors associated with movement among mobile FSWs as part of HIV prevention and access to care interventions.

## Introduction

Globally, female sex workers (FSWs) are considered a key population for the transmission and control of HIV infection (Plummer *et al*., 1991; Le *et al*., 2010; Papworth *et al*., 2013; Prüss-Ustün *et al*., 2013). A systematic review and meta-analysis of HIV burden among low- and middle-income countries suggested that FSWs are 13.5 (95% Confidence Interval (CI): 10.0–18.1) times more likely to be living with HIV than other women of reproductive age (Baral *et al*., 2012). In India, the HIV epidemic is largely concentrated in the key populations of FSWs, men who have sex with men and injecting drug users, and most HIV transmission is through heterosexual sex (87%) (NACO, 2008). Nearly two-thirds (66%) of the total HIV infection in India is reported from the states in southern (Andhra Pradesh, Karnataka and Tamil Nadu) and western (Maharashtra) India (Pandey *et al*., 2012). Of these key groups, FSWs are the most deeply affected population. According to the proximate determinant conceptual framework (Boerma & Weir, 2005), the factors associated with HIV infection among FSWs can be broadly classified into socio-demographic factors, sex work characteristics and the characteristics of agencies working on HIV prevention in these groups.

Migration for sex work is one of the key socio-demographic drivers of the geographical spread of HIV from high- to low-HIV-prevalence areas (Boerma & Weir, 2005). Migration is consistently reported as a potential driver of the HIV epidemic, and migrants (both male and female) are at increased risk of HIV infection (Lagarde *et al*., 2003; Zaba *et al*., 2005; Coffee *et al*., 2007; Rees *et al*., 2010; IOM, 2012; Reed *et al*., 2012). ‘Mobility’, in terms of short-term movements, is also a crucial factor that increases the spread of HIV infection due to the higher incidence of unsafe sex along the routes of migration (Guest, 2000). In India, most studies related to mobility and migration have revolved around employment-related male mobility. Male mobility functions as a potential bridge for the transmission of HIV infection from high- to low-risk populations along the routes of migration (Thappa *et al*., 2002; Chaturvedi *et al*., 2006; Singh *et al*., 2006; Halli *et al*., 2007; Saggurti *et al*., 2008, 2009, 2012b; Suryawanshi *et al*., 2014).

There is limited evidence in India on the movement/mobility-induced vulnerability of FSWs (Government of India, 2001; Population Council, 2008a, b, c; KHPT & Population Council, 2008; Verma *et al*., 2010; Ramesh *et al*., 2012; Saggurti *et al*., 2012a). The chance of economic improvement is a consistent motivation for movement among migrant communities across the globe, including India (Halli *et al*., 2007; Buzdugan *et al*., 2009; Saggurti *et al*., 2009). However, in the context of sex work, the reasons for mobility among FSWs are varied. Recent studies on the mobility of FSWs in southern India have indicated that high inter-state and district mobility is motivated by the need to earn more money in order to improve their economic condition and to re-pay debt (Reed *et al*., 2012). The clandestine nature of sex work is another reason for FSWs to change their sex work venues frequently. Change of place helps to avoid stigma and maintain secrecy about their work from family members (Venkataramana & Sarda, 2001).

A body of literature across the globe provides different insights and contexts associated with the mobility of FSWs. Van Blerk (2007), in his qualitative study among mobile FSWs in Ethiopia, concluded that FSWs are highly mobile in order to attract a larger or different client base, for adventure and to conceal illnesses that might be associated with AIDS. These movements of FSWs pose critical challenges, to follow-up, treatment and providing access to health services under HIV prevention programmes (Halli *et al*., 2010; Verma *et al*., 2010). Therefore, this critical aspect of migration, i.e. movement for sex work among FSWs, is of enormous importance to intervention programmes aimed at preventing and controlling the HIV epidemic. To the best of our knowledge there are no published studies on the predictors of mobility/movement among mobile FSWs and their decision to migrate/move from their current place of sex work in the Indian context. With this background, the present paper attempts to identify factors relating to the likelihood of further movement from the current place of sex work among mobile FSWs and understand the motivations behind the mobility processes in this high-risk group. The paper also explores the context of movement away from the current place of sex work and risk behaviours associated with such movement.

## Methods

### Study settings

Data were derived from a cross-sectional behavioural survey conducted among FSWs in 22 districts of high in-migration across four high-HIV-prevalence states covering the southern (Andhra Pradesh, Karnataka, Tamil Nadu) and western (Maharashtra) regions of India, as part of a study on migration/mobility and vulnerability to HIV among male migrant workers and FSWs in high-HIV-prevalence states in India, conducted from June 2007 to September 2008 (Verma *et al*., 2010). The identification of the districts was done independently on the basis of mapping and enumeration data on FSWs available from State AIDS Control Society (SACS) and the ‘Avahan’ programme’, a large-scale HIV prevention programme implemented by the Bill and Melinda Gates Foundation in 2003 in six high-HIV-prevalence states.

### Sample size, sampling design and participant recruitment

A sample size of 200 per district was pre-determined by using an estimated proportion of 30% inconsistent condom use among FSWs, an assumed difference of 3% increase in the proportion with every unit increase in degree of mobility, a confidence level of 95% and power of 80% (KHPT & Population Council, 2008; Population Council, 2008a, b, c).

In order to select FSWs from brothel and non-brothel sites (hot spots), a two-stage sampling approach was used (Verma *et al*., 2010). For selection of brothel-based FSWs, a two-stage systematic sampling technique was used with the systematic selection of the lanes/small pockets/areas within each larger brothel site. However, in the case of selection of FSWs from non-brothel areas/sites, two-stage time location sampling was used. Of 10,075 FSWs who were approached, about 94% (or 9475) agreed to respond to the screening instrument in order to participate in the study. Of these, 5611 (59%) were found to be eligible for detailed interview according to the study definition of mobile FSWs: those who had moved to two or more different locations for sex work during the previous two years, one of which was a move across districts. After dropping 113 FSWs due to refusals, withdrawal, inability to fulfil the selection criteria and missing information on socioeconomic variables, an analytical sample of 5498 FSWs remained for further analysis. Additional details of the methodology of this study are available elsewhere (KHPT & Population Council, 2008; Population Council, 2008a, b, c; Verma *et al*., 2010).

### Ethical issues

Only those FSWs who were at least 18 years of age were finally interviewed. Ethical approval for the study was obtained from the institutional review boards (IRBs) of the Population Council and the University of Manitoba, Canada. Verbal consent was obtained from all respondents prior to participation at each stage.

### Variable measures

In the bivariate and multivariate analysis, the dependent variable was ‘likelihood of further movement for sex work’, which was assessed using the question ‘Are you planning to move from this place?’ The place referred to here was the current place of sex work. The independent variables used for the predictions of likelihood of further movement for sex work were socio-demographic characteristics (current age, educational attainment, marital status and place of residence), the economic vulnerability of the respondents (income status at the current place of sex work, current debt status, debt status at the time of first move for sex work, currently under any contract) and other risk factors such as experience of physical violence as well as sexual coercion at the current place of sex work. Information about variable coding categories and their descriptions are given in Table 1.

**Table 1:** Variables used in the analysis of predictors of likelihood of further movement of FSWs in India

Variable	Coding categories	Description/definitions
**Dependent variable**		
Likelihood of further movement for sex work	0: not decided; 1: planning to move; 2: not planning to move (Ref.)	The likelihood of further movement for sex work from current place by responding to the data collection item: ‘Are you planning to move from this place?’
**Independent variables**		
*Socio-demographic*		
Current age (years)	0: ≤30 years; 1: 30+years (Ref.)	Current age in completed years
Educational attainment	0: secondary school and above; 1: up to primary school (Ref.)	Highest grade completed
Current marital status	0: unmarried; 1: formerly married; 2: married (Ref.)	Formerly married mobile FSWs include [widowed, separated, deserted and divorced]
Time in sex work (years)	0: ≤5 years; 1: 5+years (Ref.)	Computed using the difference between current age and age at starting sex work
Place of residence	1: urban; 2: rural (Ref.)	Place of interview, which is also the current place of sex work
*Economic vulnerabilities*		
Currently under contract	0: no (Ref.); 1: yes	Currently under contract for sex work with any madam or pimp
Income status at current place	0: worse; 1: better; 2: same (Ref.)	Generated using the item ‘Is your income from sex work in this city/town/place better/same/worse than in your previous place of work?’
Currently in debt	0: no (Ref.); 1: yes	Created using the question: ‘Do you owe money to anyone?’
Debt at time of first move for sex work	0: no (Ref.); 1: yes	Generated using the item ‘Did you or your family have any debt at the native place when you first moved to the sex work?’
*Sexual violence/coercion*		
Experienced violence at the current place	0: no (Ref.); 1: yes	Physical violence such as being beaten by anyone, arrested by police or thrown out of sex work place in past year
Sexual coercion at current place of work	0: no (Ref.); 1: yes	Forced sex with all types of partners (commercial or non-commercial)
Experienced anal sex at current place of sex work	0: no (Ref.); 1: yes	Generated using the question ‘What type of sexual acts do you do with this type of partner?’; those reporting ‘anal’ sex at current place (with either client or partner) are considered for the analysis
Client volume at current place of sex work	0: low (Ref.); 1: high	Number of clients (occasional or regular) served on last day at current place of sex work
HIV testing and result collection	0: done but result not collected (Ref.); 1: done and result collected	Knowledge of HIV status and collection of result
Clients’ willingness to use condoms	0: willing (Ref.); 1: unwilling	Clients’ general willingness to use condoms with FSWs. If unwilling then FSWs are more inclined to move from the place due to fear of acquisition of an STI or HIV infection. This is an indirect measure of likelihood of movement from current place of sex work.

To assess HIV risk behaviours such as consistent condom use with different types of clients or partners (defined as those reporting ‘always’ when asked about the frequency of using condoms), forced sex with clients or partners (defined as those reporting ‘yes’ to the question ‘Was there any time that these partners beat/physically force you to have sex?’), consistent use of alcohol prior to sex of clients and partners (defined as those reporting ‘always’ when asked about the frequency of indulging in alcohol prior to sex), self-perceived HIV risk, STI symptoms (those reporting either of any symptoms like ulcers/sores in genital area, swelling in groin area, pain during intercourse and frequent painful urination in the past six months) and STI risk (i.e. continued to have sex despite having STI symptoms), the key independent variable was ‘likelihood of further movement for sex work’, with other socio-demographic variables being control variables.

### Statistical analyses

Univariate analysis was carried out to calculate percentages and summary measures like median and inter-quartile range to describe the profile of the mobile FSWs among different high-HIV-prevalence states. Bivariate analysis was performed to assess the association between the key dependent variable, i.e. likelihood of further movement for sex work from current place, and independent variables and HIV risk behaviours using chi-squared test statistics. Multinomial logistic regression models were constructed to examine the predictors of likelihood of further movement from current place of sex work relative to the population of FSWs who were not planning to move and who were indecisive about the movement from their current place of work. Measures such as age, educational attainment, current marital status, time in sex work (in years), experience of physical violence, sexual coercion and income status at the current place of sex work, current status of debt, debt status at the time of first move for sex work, currently under contract and place of residence were controlled during the construction of the multivariate logistic regression models. The results of the multinomial logistic regression model are presented in the form of adjusted odds ratios, along with the corresponding 95% confidence interval (95% CI). A series of multiple logistic regression models were run to assess the effect of further planning to move from current place of sex work on HIV risk behaviours. A *p-*value of 5% was considered significant, and all analyses were done using SPSS 18.0.

## Results

Of the sampled population of mobile FSWs, nearly two-thirds (64%) were in the younger age group, i.e. less than 30 years of age, with the highest proportion being from Karanataka State (93%). The median age was 29 years (IQR=8 years) and the median time in sex work was 5 years (IQR=5 years) (Table 2). More than half (52%) of the mobile FSWs had attained an educational level of up to primary school, with a higher proportion of them being in Maharashtra (65%). One-third (34%) were currently married, and only 15% were unmarried. The proportion formerly married was more than half (52%). Over half (57%) reported a ‘better’ income status at their current place of work and more than one-third (37%) reported no change in income status at their current place compared with their previous place of sex work. More than two-fifths (46%) reported being currently in debt and more than one-third (35%) were in debt at the time of their first move for sex work. Only 10% were under contract and most of them were residing in urban areas (85%). About one-fifth (22%) of the FSWs had experienced violence at their current place of sex work and a similar proportion reported sexual coercion (21%) and anal sex being demanded by clients (19%). Forty per cent of mobile FSWs reported that clients were unwilling to use condoms during sexual acts. Although all mobile FSWs admitted to having their HIV test done, a majority (three-fifths or 60%) had not collected the test result.

**Table 2:** Profile of mobile FSWs in the study’s four high-HIV-prevalence Indian states (*N*=5498)

	Andhra Pradesh (*N*=1533)	Karanataka (*N*=1500)	Maharashtra (*N*=1189)	Tamil Nadu (*N*=1276)	Total (*N*=5498)
Background characteristics	% (*n*)	% (*n*)	% (*n*)	% (*n*)	% (*n*)
Current age (years)					
≤30	58.2 (892)	92.7 (1390)	68.5 (814)	34.6 (442)	64.4 (3538)
31+	41.8 (641)	7.3 (110)	31.5 (375)	65.4 (834)	35.6 (1960)
Median age (IQR)	30 (9)	27 (4)	29 (8)	35 (10)	29 (8)
Educational attainment					
Secondary school and above	40.4 (620)	42.3 (635)	35.1 (417)	74.3 (948)	47.7 (2620)
Up to primary school	59.6 (913)	57.7 (865)	64.9 (772)	25.7 (328)	52.3 (2878)
Current marital status					
Unmarried	8.7 (134)	25.4 (381)	22.6 (269)	0.9 (11)	14.5 (795)
Formerly married	57.4 (880)	64.1 (961)	51.4 (611)	31.5 (402)	51.9 (2854)
Currently married	33.9 (519)	10.5 (158)	26.0 (309)	67.6 (863)	33.6 (1849)
Time in sex work (years)					
≤5	54.1 (829)	88.7 (1330)	54.3 (646)	45.8 (584)	61.6 (3389)
6+	45.9 (704)	11.3 (170)	45.7 (543)	54.2 (692)	38.4 (2109)
Median (IQR)	5 (7)	3 (2)	5 (6)	6 (6)	5 (5)
Current place of residence					
Urban	75.8 (1162)	98.3 (1474)	82.7 (983)	84.2 (1075)	85.4 (4694)
Rural	24.2 (371)	1.7 (26)	17.3 (206)	15.8 (201)	14.6 (804)
Currently under contract	11.9 (182)	16.2 (243)	6.1 (73)	2.0 (26)	9.5 (524)
Income from the current place					
Worse	4.6 (71)	5.9 (89)	6.4 (76)	6.6 (84)	5.8 (320)
Better	61.7 (946)	64.5 (967)	38.1 (453)	61.5 (785)	57.3 (3151)
Same	33.7 (516)	29.6 (444)	55.5 (660)	31.9 (407)	36.9 (2027)
Currently in debt	70.4 (1079)	33.3 (499)	31.0 (369)	43.3 (553)	45.5 (2500)
Debt at time of first move for sex work	47.6 (730)	34.9 (524)	32.9 (391)	22.2 (283)	35.1 (1928)
Client volume at current place of sex work					
Low	39.4 (604)	67.1 (1007)	29.9 (355)	62.7 (800)	50.3 (2766)
High	60.6 (929)	32.9 (493)	70.1 (834)	37.3 (476)	49.7 (2732)
Experienced physical violence at current place of sex work	32.8 (503)	24.1 (362)	14.1 (168)	14.0 (179)	22.0 (1212)
Sexual coercion	24.7 (379)	28.7 (431)	22.6 (269)	7.2 (92)	21.3 (1171)
Experienced anal sex	36.2 (555)	15.5 (233)	9.9 (118)	10.7 (137)	19.0 (1043)
Clients’ willingness to use condoms					
Willing	68.3 (1047)	23.1 (346)	74.3 (884)	79.2 (1010)	59.8 (3287)
Unwilling	31.7 (486)	76.9 (1154)	25.7 (305)	20.8 (266)	40.2 (2211)
HIV testing/result collection^a^					
HIV testing done; result not collected	34.6 (507)	42.2 (313)	77.3 (819)	85.0 (1026)	59.6 (2665)
HIV testing done; result collected	65.4 (960)	57.8 (429)	22.7 (240)	15.0 (181)	40.4 (1810)

a Among those who had ever had HIV testing done (*N*=4475).

### Predictors of likelihood of further movement from current place of sex work

The interpretation of the predictors of further movement of mobile FSWs is given in two sections as the outcome variable has three categories and multinomial logistic regression provides a comparison in two different sets by keeping one category relative (i.e. the reference category) and other two as test categories. In this analysis, ‘Not planning to move’ is the relative/reference category.

#### Not decided vs not planning to move

Table 3 presents the multinomial logit odds of predictor variables for predicting indecision about moving to another place of sex work relative to not planning to move from the current place of sex work among mobile FSWs. Compared with non-contracted mobile FSWs, contracted mobile FSWs were more likely to be indecisive about moving to a further place of sex work relative to those who were not planning to move (aOR: 1.36; 95% CI: 1.01–1.84). Similarly, compared with their reference categories, mobile FSWs with a better income at their current place of work (aOR: 1.38; 95% CI: 1.17–1.63) and having had an HIV test and collected the result (aOR: 1.66; 95% CI: 1.39–1.99) were more likely to be indecisive about their plan to move from their current place of sex work relative to mobile FSWs those who were not planning to move.

**Table 3:** Multivariate multinomial logistic regression modelling predicting the outcome variable (likelihood of further movement for sex work) among mobile FSWs, India (*N*=5498): not decided vs not planning to move (Reference category)

							95% CI for Exp(*B*)	
Predictor variables	*B*	SE	Wald	df	Sig.	Exp(*B*)	Lower	Upper
Intercept	−0.539	0.16	11	1	0.001			
Age ≤30 years (Ref.:30+years)	0.041	0.09	0.2	1	0.648	1.04	0.87	1.24
Education: up to primary school (Ref.: secondary school and above)	0.112	0.08	1.8	1	0.183	1.12	0.95	1.32
Unmarried (Ref.: currently married)	0.178	0.16	1.2	1	0.270	1.19	0.87	1.64
Formerly married (Ref.: currently married)	−0.114	0.09	1.7	1	0.193	0.89	0.75	1.06
Time in sex work: ≤5 years (Ref.: 5+ years)	−0.101	0.09	1.4	1	0.238	0.90	0.76	1.07
Andhra Pradesh (Ref.: Tamil Nadu)	1.694	0.12	195	1	0.000	5.44	4.29	6.90
Karnataka (Ref.: Tamil Nadu)	2.526	0.17	221.7	1	0.000	12.51	8.97	17.44
Maharashtra (Ref.: Tamil Nadu)	2.943	0.14	426.5	1	0.000	18.97	14.35	25.08
Place of residence: urban (Ref.: rural)	−0.199	0.1	3.7	1	0.055	0.82	0.67	1.00
Under contract: yes (Ref.: no)	0.309	0.15	4.1	1	0.044	1.36	1.01	1.84
Income at current place: worse (Ref.: same)	−0.484	0.17	7.9	1	0.005	0.62	0.44	0.86
Income at current place: better (Ref.: same)	0.323	0.08	14.9	1	0.000	1.38	1.17	1.63
Currently in debt: yes (Ref.: no)	−0.218	0.08	6.7	1	0.01	0.80	0.68	0.95
Debt at the time of first move for sex work: yes (Ref.: no)	−0.643	0.09	54.5	1	0.000	0.53	0.44	0.62
Experienced physical violence at current place: yes (Ref.: no)	−0.062	0.1	0.4	1	0.534	0.94	0.77	1.14
Experienced sexual coercion at current place: yes (Ref.: no)	0.132	0.11	1.5	1	0.22	1.14	0.92	1.41
Experienced anal sex at current place: yes (Ref.: no)	−0.608	0.1	37.3	1	0.000	0.54	0.45	0.66
Client volume at current place: low (Ref.: high)	−0.011	0.08	0	1	0.896	0.99	0.84	1.16
HIV testing and result collection: yes (Ref.: no)	0.508	0.09	30.5	1	0.000	1.66	1.39	1.99
Clients’ willingness to use condoms: no (Ref.: yes)	−0.058	0.09	0.4	1	0.529	0.94	0.79	1.13

Pseudo *R* ^2^: Cox and Snell: 0.29; Nagelkerke: 0.35; McFadden: 0.19.

Additionally, compared with their counterparts, mobile FSWs with a ‘worse’ income at their current place of work (aOR: 0.62; 95% CI: 0.44–0.86), under debt (aOR: 0.80; 95% CI: 0.68–0.95), under debt at the time of first move for sex work (aOR: 0.53; 95% CI: 0.44–0.62) and who experienced anal sex at their current place of work (aOR: 0.54; 95% CI: 0.45–0.66) were less likely to be indecisive about moving again relative to the population of FSWs who were not planning to move.

#### Planning to move vs not planning to move

Table 4 presents the multinomial logit odds of predictor variables for predicting the decision about planning to move to another place of sex work relative to not planning to move among mobile FSWs. Compared with the currently married, unmarried mobile FSWs were more likely to plan to move again from their current place of sex work relative to the population of mobile FSWs who were not planning to move (aOR: 1.76; 95% CI: 1.11–2.80).

**Table 4:** Multivariate multinomial logistic regression modelling predicting the outcome variable (likelihood of further movement for sex work) among mobile FSWs, India (*N*=5498): planning to move vs not planning to move (reference category)

							95% CI for Exp(*B*)	
Predictor variables	*B*	SE	Wald	df	Sig.	Exp(*B*)	Lower	Upper
Intercept	−4.363	0.34	164.8	1	0.000			
Age ≤30 years (Ref.: 30+years)	0.034	0.16	0.0	1	0.830	1.03	0.76	1.41
Education: up to primary school (Ref.: secondary school and above)	0.407	0.14	9.1	1	0.003	1.50	1.15	1.96
Unmarried (Ref.: currently married)	0.568	0.24	5.8	1	0.016	1.76	1.11	2.80
Formerly married (Ref.: currently married)	0.099	0.16	0.4	1	0.536	1.10	0.81	1.51
Time in sex work: ≤5 years (Ref.: 5+ years)	0.181	0.15	1.5	1	0.225	1.20	0.89	1.61
Andhra Pradesh (Ref.: Tamil Nadu)	1.170	0.26	20.3	1	0.000	3.22	1.94	5.36
Karnataka (Ref.: Tamil Nadu)	3.510	0.29	142.4	1	0.000	33.44	18.79	59.52
Maharashtra (Ref.: Tamil Nadu)	3.350	0.27	154.3	1	0.000	28.51	16.81	48.37
Place of residence: urban (Ref.: rural)	0.124	0.21	0.4	1	0.551	1.13	0.75	1.70
Under contract: yes (Ref.: no)	0.369	0.21	3.1	1	0.076	1.45	0.96	2.17
Income at current place: worse (Ref.: Same)	−0.158	0.31	0.3	1	0.610	0.85	0.47	1.56
Income at current place: better (Ref.: same)	0.593	0.15	16.4	1	0.000	1.81	1.36	2.41
Currently in debt: yes (Ref.: no)	0.593	0.14	17.9	1	0.000	1.81	1.38	2.38
Debt at the time of first move for sex work: yes (Ref.: no)	−0.943	0.14	43.2	1	0.000	0.39	0.29	0.52
Experienced physical violence at current place: yes (Ref.: no)	0.572	0.16	13.4	1	0.000	1.77	1.30	2.41
Experienced sexual coercion at current place: yes (Ref.: no)	0.355	0.17	4.6	1	0.031	1.43	1.03	1.97
Experienced anal sex at current place: yes (Ref.: no)	−0.153	0.16	0.9	1	0.344	0.86	0.62	1.18
Client volume at current place: low (Ref.: high)	−0.576	0.14	17.3	1	0.000	0.56	0.43	0.74
HIV testing and result collection: yes (Ref.: no)	1.166	0.15	62.0	1	0.000	3.21	2.40	4.29
Clients’ willingness to use condoms: no (Ref.: yes)	−0.075	0.15	0.3	1	0.616	0.93	0.69	1.24

Pseudo *R* ^2^: Cox and Snell: 0.29; Nagelkerke: 0.35; McFadden: 0.19.

Similarly, compared with their counterparts, mobile FSWs who had completed secondary school and above education (aOR: 1.50; 95% CI: 1.15–1.96), were currently in debt (aOR: 1.81; 95% CI: 1.38–2.38), experienced physical violence at their current place of work (aOR: 1.77; 95% CI: 1.30–2.41), experienced sexual coercion at their current place of work (aOR: 1.43; 95% CI: 1.03–1.97) and those who had HIV testing done and collected the result (aOR: 3.21; 95% CI: 2.40–4.29) were more likely to plan to move from their current place of sex work relative to the population of mobile FSWs who were not planning to move. Moreover, compared with mobile FSWs with ‘high’ client volume at their current place of work, ‘low’ client volume FSWs (aOR: 0.56; 95% CI: 0.43–0.74) were less likely to plan to move from their current place of sex work relative to those not planning to move. The post-hoc analysis suggests that the ‘age’ factor plays a crucial role in the lower propensity of movement from current place of work, despite there being a low client volume among this subgroup of mobile FSWs. The older mobile FSWs among this subgroup were less likely to move from their current place of work compared with young mobile FSWs. This is supported by the bivariate analysis of age and the outcome variable ‘planning to move’. The proportion of younger mobile FSWs (12% vs 6%, *p*<0.0001) planning to move from their current place of work was higher than that of older mobile FSWs.

### HIV-related risk behaviours in relation to likelihood of further movement from current place of work

#### Condom use

Consistent condom use with occasional (aOR: 0.64; 95% CI: 0.48–0.86) and regular clients (aOR: 0.40; 95% CI: 0.30–0.52) among mobile FSWs who were planning to move from their current place of work was less than that of their counterparts who were not planning to move (Table 5). A similar finding was observed when a comparison was made between the group of mobile FSWs who were indecisive about their further movement and those not planning to move from their current place of sex work. The odds of condom use at the time of forced sex with an occasional (aOR: 2.21; 95% CI: 1.11–4.38) or regular client (aOR: 2.53; 95% CI: 1.19–5.40) were higher among mobile FSWs who were planning to move compared with those who were not planning to move.

**Table 5:** HIV-related risk behaviours reported by mobile FSWs by their likelihood of planning to move from current place of sex work (*N*=5498)

	Planning to move further for sex work				
	Not decided (%)	Planning to move (%)	Not planning to move (%)	Not decided vs not planning to move (Ref.)	Planning to move vs not planning to move (Ref.)
HIV risk behaviours	%	%	%	aOR (95% CI)	aOR^a^ (95% CI)
Consistent condom use					
Occasional clients	65.8	55.9	83.0	0.60 (0.49–0.72)	0.64 (0.48–0.86)
Regular clients	55.7	38.4	76.7	0.63 (0.53–0.75)	0.40 (0.30–0.52)
Other non-paying partners	40.9	26.9	57.4	1.16 (0.88–1.53)	0.72 (0.47–1.10)
Consistent alcohol use prior to sex					
Occasional clients	21.5	27.8	20.9	1.17 (0.99–1.38)	1.60 (1.25–2.06)
Regular clients	22.4	28.2	29.6	1.08 (0.92–1.27)	1.45 (1.13–1.86)
Other non-paying partners	30.8	18.4	34.3	0.81 (0.62–1.05)	0.42 (0.27–0.65)
Forced sex/sexual coercion					
Occasional clients	16.1	27.6	11.0	1.08 (0.88–1.32)	1.98 (1.51–2.60)
Regular clients	11.5	19.8	8.7	1.10 (0.87–1.38)	2.00 (1.47–2.72)
Other non-paying partners	15.2	23.4	15.7	0.59 (0.42–0.82)	0.87 (0.55–1.38)
Any sex partner	22.1	33	15.7	1.08 (0.90–1.29)	1.75 (1.37–2.24)
Condom use at time of forced sex					
Occasional clients	32.6	28.9	21.0	2.60 (1.54–4.38)	2.21 (1.11–4.38)
Regular clients	19.9	25.7	25.0	1.32 (0.75–2.33)	2.53 (1.19–5.40)
Other non-paying partners	28.1	21.3	19.3	2.57 (1.10–6.04)	1.20 (0.38–3.73)
Alcohol use prior to sex by FSW with any partner	12.1	14.4	14.0	0.86 (0.70–1.05)	1.00 (0.73–1.36)
Have STI symptoms	72.5	82.3	65.3	1.13 (0.96–1.31)	1.51 (1.14–2.00)
Continued to have sex despite having STI symptoms (STI risk)	21.0	28.0	19.8	0.59 (0.50–0.70)	0.86 (0.67–1.10)
Self-perception of HIV risk	36.8	55.9	41.3	0.83 (0.72–0.96)	1.87 (1.49–2.35)

a Controlled for current age, educational attainment, marital status, income at current place, time in sex work, state, currently under contract, place of residence and currently in debt.

aOR: adjusted odds ratio.

#### Sexual coercion or forced sex

The odds of sexual coercion or forced sex were higher among mobile FSWs who were planning to move compared with those who were not planning to move. This was true for both types of commercial partners: occasional clients (aOR: 1.98; 95% CI: 1.51–2.60) and regular clients (aOR: 2.00; 95% CI: 1.47–2.72). However, in the case of other non-paying partners (i.e. non-commercial partners) the odds of forced sex were lower among mobile FSWs who were indecisive about movement compared with those not planning to move (aOR: 0.59; 95% CI: 0.42–0.82). When sexual coercion irrespective of partner type was assessed, it was found that it was greater among mobile FSWs planning to move compared with their counterparts for any type of partner (aOR: 1.75; 95% CI: 1.37–2.24).

#### Alcohol use prior to sex

The odds of consistent alcohol use prior to sex by occasional (aOR: 1.60; 95% CI: 1.2–2.06) and regular clients (aOR: 1.45; 95% CI: 1.13–1.86) were greater among mobile FSWs planning to move from their current place of work compared with their counterparts. In contrast, the odds of consistent alcohol use prior to sex by non-paying partners (aOR: 0.42; 95% CI: 0.27–0.65) were lower among mobile FSWs planning to move compared with those not planning to move.

#### STI symptoms and risk and self-perceived risk of HIV infection

The odds of having STI symptoms (aOR: 1.51; 95% CI: 1.14–2.00) and the self-perceived risk of HIV infection (aOR: 1.87; 95% 1.49–2.35) were higher among mobile FSWs planning to move from the current place of work compared with their counterparts who were not planning to move. Additionally, the odds of STI risk (aOR: 0.59; 95% CI: 0.50–0.70) were lower among FSWs who were indecisive about their movement compared with those not planning to move from their current place of sex work.

## Discussion

Very few studies have documented the motivations behind the movement of mobile FSWs. The most common reported motivations are higher earning opportunity and economic improvement. This cross-sectional study provides critical evidence on the predictors of the likelihood of further movement of mobile FSWs from their current place of sex work. The findings suggest that socioeconomic as well as sexual behavioural factors play a role in the continual movement of mobile FSWs.

Higher educational attainment was found to be a predictor of the further movement of mobile FSWs from their current place of sex work. In the general population, educational attainment plays a primary role in mobility as it increases earning potential (Wolbers, 2000). However, in the context of the sex work industry, the association between educational attainment and the likelihood of further movement from current place of sex work is an unexpected finding and requires more research. Another demographic characteristic that emerged as a crucial predictor of the decision to move from the current place of sex work was marital status. Unmarried FSWs are more likely to plan to move than married ones. This could be due to their relative independence in terms of decision-making, as they do not have the responsibility of children or spouses. The literature on gender-based mobility suggests that women with childcare and other household responsibilities are less mobile compared with unmarried or formerly married woman. This was validated in a study by Banerjee & Raju (2009), who concluded that marital status does not constrain men as much as it does married women since childcare and care of the elderly keep women from joining, or continuing in, the formal labour market. Restriction on women’s mobility due to domestic workload is common in many countries, and limits their ability to participate in community or market-related activities (Upadhyay, 2010; Njuki *et al*., 2013). This seems to be true of FSWs as well.

This study found sex work under the contractual system to be a strong driving force for further movement among FSWs. Almost half of the mobile FSWs who were under the contractual system planned to move from their current place of sex work compared with those who were not under contract. Female sex workers under a contractual system are controlled by contractors who can force them to move, but those working under brothel madams or other third parties have restrictions imposed on them (Sen & Nair, 2004; Gupta *et al*., 2009). George *et al*. (2011) suggested that FSWs who go on contract work may be more financially vulnerable than those who work in their home districts. Studies among mobile FSWs have indicated high levels of debt (Population Council, 2008a, b, c; KHPT & Population Council, 2008), which in turn lead to financial vulnerability, engagement in riskier contract work that compromises their ability to demand or negotiate safer sexual practices, and a less safe work environment. This was validated by Bharat *et al* (2013), who concluded that not being under contract allows mobile FSWs to negotiate safer sex practices in terms of condom use, even in new places of sex work.

It is consistently reported that economic condition is a push factor for entry into sex work (Saggurti *et al*., 2011b). In order to overcome economic condition and re-pay debt, FSWs keep moving to earn more money by seeking a new and larger client base (Reed *et al*., 2012). This was confirmed in the present study. Debt and poor income status at their current place of work were found to be significant predictors of the likelihood of planning to move to a new place of sex work. Further, experience of violence and sexual coercion at their current place of sex work was also a significant predictor of FSW mobility. This has been cited in other studies in India and elsewhere as a reason for mobility of FSWs and increased HIV vulnerability (WHO, 2005; Jewkes *et al*., 2010; Wang *et al*., 2010; Ramesh *et al*., 2012; Saggurti *et al*., 2012a). Female sex workers forced to work in new environments with unknown clients are more vulnerable (KHPT & Population Council, 2008; Beattie *et al*., 2010). The lack of community ties for social support enhances their vulnerability to HIV and endangers their personal safety (Van Blerk, 2007).

An unexpected finding of this study was that those who had been tested for HIV and collected their test result were more likely to plan to move again compared with those who had tested but not collected their result. It is possible that the HIV test reports for these FSWs were positive, and hence fear of discrimination and stigma, and the need for secrecy about their HIV status among male clients, motivated them to move. Previous studies among HIV-positive women in southern India have found that non-disclosure of HIV status is related to negative outcomes such as greater fear of stigma and discrimination, and a sense of futility (Chandra *et al*., 2003). Additionally, Saggurti *et al* (2013a) reported that among HIV-positive FSWs non-disclosure of HIV status to male clients is due to a fear of losing clientele and business.

This study found that mobile FSWs who were planning to move from their current place of work had higher levels of HIV-related risk compared with those who were not planning to move or who were undecided about movement. They reported low levels of consistent condom use with clients (i.e. during paid sex), thus potentially transmitting HIV and other sexual infections across the places they visit. Reasons for low levels of consistent condom use were reported to be poor economic conditions (FSWs make more money when condoms are not used), low level of condom negotiation skills due to the newer environment and new work place, violence and forced sex (WHO, 2005; Ntumbanzondo *et al*., 2006; Silverman *et al*., 2007; Bharat *et al*., 2013). Additionally, those mobile FSWs who planned to move were more likely to report alcohol use by clients prior to sex than their counterparts who did not plan to move. This behaviour of male clients has been reported to result in unprotected sex with FSWs, placing both at increased risk of HIV acquisition (Verma *et al*., 2010). Moreover, sexual coercion plays a double role as a ‘predictor’ of further movement as well as of HIV risk behaviour among this sub-group of mobile FSWs. This might be due to the new workplace/environment and lack of social support from the community networks of local FSWs (Swain *et al*., 2011). Female sex workers who had plans to move again from their current place of sex work also reported more STI symptoms and a high level of risk perception to HIV. The reason for this could be their inability to access local health services or non-exposure to HIV prevention programmes due to continuous movement from one place to another. This suggests that the decision about movement from current place of work does not change their riskier behaviour. However, inconsistent condom use, alcohol use prior to sex and sexual coercion by commercial partners (occasional and regular clients) increases the likelihood of STI risk and self-perceived HIV risk among mobile FSWs, and hence increases the likelihood of further movement from their current place of work.

The findings discussed here have important programmatic implications, but must be interpreted in light of certain limitations. Firstly, the key independent variables considered were based on self-reported responses, which are subject to social desirability and recall bias. Secondly, the study did not use a comparative research design to include non-mobile FSWs; hence the findings apply only to those FSWs who moved and not to the general community of FSWs. Thirdly, this study suggests that socioeconomic and behavioural vulnerabilities increase the chances of acquiring HIV infection among mobile FSWs, and also among non-mobile FSWs. However, due to non-inclusion of non-mobile FSWs in the study design, these effects could not be confirmed from this current study. Despite careful consideration of the reference period and multiple questions about consistent condom use and STI symptoms, the bias in self-reported responses cannot be completely reduced, and hence the results must be interpreted with caution (Saggurti *et al*., 2011a).

Some of the contextual or structural factors such as economic need and debt are consistently reported as primary reasons for movement from the current place of sex work among mobile FSWs. This study further validates this critical evidence along with sexual behavioural factors such as sexual coercion, violence at the current place and HIV risk perception. These individual-level structural factors, along with the behavioural factors, increase the chances of further movement among mobile FSWs and support the indirect demand for creating an enabling environment for these high-risk group of mobile FSWs. The lack of social support, newer work environment and desire to earn more by searching for a high client volume at new places, or the competition to acquire more clients, appear to keep FSWs highly mobile and increase the likelihood of further movement. This mobility intensifies their vulnerability in terms of low level of consistent condom use, exposure to STI risk and high perceived risk of HIV acquisition. These determinants and risky behaviours were not only observed among those groups of FSWs who were planning to move, but also among those indecisive about their plans for further movement. This creates an urgent need for HIV prevention programme designers to innovate strategies to reach out to mobile FSWs at their new places of work. One such strategy is the formation of community networks of sex workers that mobile FSWs could access at their new locations. Such networks would meet their need for condoms and support them in times of crisis due to physical or sexual violence by sexual partners, clients and others (police and local ‘goons’). Recent studies in India and elsewhere suggest that such community networks can be successful in mobilizing FSW communities to reduce their vulnerability to HIV and physical or sexual violence (Gaikwad *et al*., 2012; Guha *et al*., 2012; Reza-Paul *et al*., 2012; Saggurti *et al*., 2013b; Moore *et al*., 2014).
